# The management of personal protective equipment during the COVID-19 pandemic: The case of the province of Quebec

**DOI:** 10.1177/08404704211053996

**Published:** 2022-01-28

**Authors:** Martin Beaulieu, Jacques Roy, Claudia Rebolledo, Sylvain Landry

**Affiliations:** 1 200307 HEC Montréal, Montreal, Quebec, Canada.; 2 10014 HEC Montréal, Montreal, Quebec, Canada.

## Abstract

Like other Canadian provinces, Quebec managed shortages of Personal Protective Equipment (PPE) in the early weeks of the COVID-19 pandemic. Two years later, with hindsight, what lessons can we learn from this logistics crisis? It is precisely to better understand the decisions made by the supply chain players in the province of Quebec that this paper was written. To fully understand the events, this paper first describes the Quebec healthcare system. Then it retraces the series of events and actions during the first wave, at a time when it was most challenging to procure PPE. It also specifies the main characteristics of the supply chain in the Quebec healthcare sector. Finally, it analyzed these data to come up with recommendations to help public decision makers adopt better supply chain management practices.

## Introduction

The supply of Personal Protective Equipment (PPE) was one of the most critical issues in the first wave of the COVID-19 pandemic. In a race against the clock, most of the industrialized countries were searching for the same products to meet increasingly desperate needs. But now, two years later, with hindsight, what lessons can we learn from this logistics crisis? It is precisely to better understand the decisions made by the supply chain players in the province of Quebec that this paper was written. This case made up part of a national CIHR Rapid Research Grant (# CIHR Ref. VR5 172669) entitled “Development of an Implementation Framework to Advance Provincial and National Health System Supply Chain Management of the COVID-19 Pandemic.”

Before presenting our recommendations, we first describe the methodology of our study and the Quebec healthcare system. Then we chart the series of events and actions of the first wave, when it was most challenging to procure PPE. Finally, we analyze these data to come up with recommendations to help public decision makers adopt better supply chain management practices.

## Methodology

The case is built on interviews of supply chain managers from eight Quebec hospitals, selected to reflect the diversity of healthcare institutions in the province and regional differences in the pandemic’s impact. A purposive sampling was used to obtain a diversity of respondents following some relevant criteria. For example, we wanted to have respondents from regions harshly affected by the first wave of COVID-19. We also wanted to get information from hospitals with different missions: short-term hospitals, long-term care centres, and university hospitals. The eight selected hospitals cover almost one quarter of all establishments in the province. The interview guide intended to collect information about the organizational structure of the hospital and its logistics department, during and after the first wave of the pandemic. We also interviewed two PPE suppliers and several managers from Quebec’s Ministry of Health and Social Services logistics directorate and from the healthcare purchasing group. These last two organizations played a central role in the provincial management of PPE. In total, more than 20 semi-structured interviews, lasting 60 to 120 minutes each, were conducted between September 2020 and February 2021.

### Quebec’s healthcare system^1^

**Figure 1.**
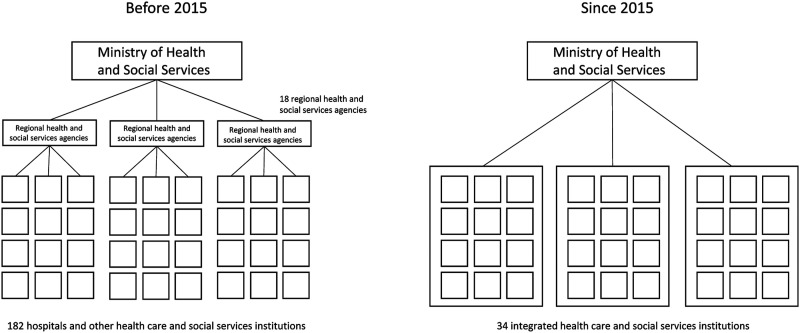
Quebec’s healthcare sector structure before and since the 2015 reform.

In the province of Quebec, a reform carried out in the spring of 2015 decreased the number of public health institutions from 182 to 34. Figure 1 illustrates the major changes made by the reform. Prior to that time, there had been regional health and social service agencies in place to coordinate and develop the healthcare offering within each region’s healthcare institutions. These agencies provided coordination at the intersection point between the institutions themselves and the guidance given by the provincial Ministry of Health and Social Services. But this level of governance was eliminated during the reform. Now, institutions are directly accountable to the Ministry. These new consolidated institutions are similar to the Integrated Delivery Networks (IDNs) model that appeared in the United States in the late 1990s, whose goal was to offer a wide range of health services under a single administration in order to facilitate the patient’s care trajectory.^2^ When presenting his reform, the minister mentioned explicitly the Kaiser Permanente model as one of the most performant IDN in the United States.^3^

Quebec’s new institutions not only manage short-term care hospitals but also long-term care centres and community services. This can result in institutions consisting of about 100 facilities spread over an area of thousands of square kilometres. The network of public long-term care hospitals is completed with private-unfunded and private-funded institutions. This last category is made up of private organizations that have a service agreement with the regional institution to offer “beds” and long-term care, often for semi-autonomous patients.

Unlike most other provinces in the country, Quebec’s Ministry of Health and Social Services has its own logistics directorate. The traditional role of this directorate is to coordinate procurement and logistics initiatives between institutions within the system or between these facilities and other parts of the government. The directorate does not carry out operational activities such as product or service contracting or inventory management. Rather, its primary role is to provide guidance on supply chain issues. While the role of logistics has progressed incrementally in the Quebec health system since the 2000s,^4^ the spring 2015 reform created the conditions for revaluating healthcare logistics in institutions. The reform has been accompanied by the creation of logistics departments, and a logistics director now sits on each board of directors.

One feature of Quebec’s healthcare sector had been the presence of three purchasing groups that handled the contracting of about 30% of the healthcare institutions’ procurement budget.^5^ As with other commodities in the healthcare sector, contracts for PPE in Quebec are very often awarded through group purchasing organizations that leverage the consolidated volumes from their member institutions to obtain low prices for these items.^6^ The use of group purchasing organizations puts pressure on suppliers to stay competitive to obtain or retain a significant market share. This phenomenon is not unique to Quebec as group purchasing organizations are found elsewhere in Canada and in the United States.^7,8^ It should be noted that, in winter 2019, a further Quebec government reform merged the three healthcare purchasing groups into a single organization that would no longer be accountable to the Ministry of Health and Social Services but instead to the Treasury Board. This new organization was to enter into service on April 1, 2020, but this was pushed back to September 1, 2020, due to the pandemic.

## Key dates

To provide a timeline of the decisions made by the main players in the Quebec healthcare sector, Table 1 outlines the major events in the PPE crisis. This table places the key decisions related to PPE management in the timeline of the major events of the pandemic.

**Table 1.** Timeline of major events.

Date	Event
2019-12-01	Patient zero (unofficial) identified in China’s Wuhan region
2019-12-31	China reveals it has identified a new coronavirus strain
Early 2020	Healthcare suppliers with international operations pick up signals from their offices in Asia that some troubling things are happening in China
Mid-January 2020	One hospital in the province of Quebec places a large PPE order, equivalent to six months’ worth of normal consumption
2020-01-23	Public health publishes an internal Ministry of Health report on the coronavirus
2020-01-24	Start of the Chinese New Year festivities, and strict lockdown of the Wuhan region until April 8, 2020
2020-02-05	First meeting of a tactical committee on PPE, led by the logistics directorate of Quebec’s Ministry of Health and Social Services. This committee included PPE providers, among others
2020-02-27	The province’s first COVID-19 case is confirmed
2020-03-13	Quebec declares a public health emergency. The Minister of Health and Social Services and the health and social services institutions may, without delay and without formality […], enter into new contracts as they deem necessary
2020-03-16	The first COVID-related death occurs in the province of Quebec
2020-03-20	The Ministry sends a letter to all 34 institutions stating that they can no longer order from PPE suppliers and that the Ministry will allocate supplies. The Ministry becomes the intermediary between the needs of institutions and the offer from the supplier market. The 34 institutions then become hubs responsible for redistributing PPE to other public institutions (schools, daycare centres, and prisons) and senior residences
2020-03-31	In a press conference, the Quebec premier acknowledges that a shortage of some equipment, particularly masks, threatens to hit Quebec within 3 to 5 days
Mid-April 2020	The provincial stockpile has a growing inventory
2020-04-22	Quebec exceeds 20 COVID-19 deaths per 100,000 inhabitants
2020-05-17	Quebec exceeds 50 COVID-19 deaths per 100,000 inhabitants
2020-06-29	The allocation strategy is modified, moving from a centralized management of PPE supplier relationships and returning to decentralized order management by healthcare institutions

Abbreviation: PPE, personal protective equipment.

The timeline of events shows that, long before the COVID-19 pandemic struck Quebec, there was a logistics crisis in the supply of PPE. Unlike in Europe or the United States, where 50% of PPE needs are generally met by local production,^9^ Canada was almost entirely dependent on foreign markets for these items prior to the COVID-19 pandemic.^10^ The characteristics of the PPE supply chain (low-cost strategy and production offshored to Asia) had led to China, and specifically the Wuhan region, becoming the world’s main PPE production centre.^11^ The first impacts of the pandemic arrived just at the start of the Chinese New Year festivities on January 24, 2020, and the subsequent four-week interruption in China’s economic activity. Also, the Wuhan area was placed under a strict lockdown to stop the spread of the virus. China’s reduced activity during this time meant that global PPE production capacity was significantly truncated for over two months.^12^ Under these specific circumstances, the window of opportunity for securing an inventory of PPE had become very narrow.

Starting in early February 2020, the Health Ministry’s logistics directorate established a PPE crisis unit, which became the control centre for provincial needs and the offer available on the supply market. This unit began with just a few individuals, but it incorporated other employees from the health system (institutions and purchasing groups) as new needs or problems arose, and it eventually became the COVID-19 operations team. Up to 60 people were directly involved in this committee, which had regular contact with the industry’s major suppliers. Its mandate was initially focused on PPE management, but it eventually also took on needs relating to screening tests and to the purchase of treatment equipment, such as respirators, for the hospitals.

## The state of the supply chain infrastructure before the crisis

After conducting interviews with stakeholders, we identified four basic features that undermined the efficiency of the PPE procurement process during the pandemic. First, relations between the Health Ministry’s logistics directorate and the 34 healthcare institutions had historically been consensus based. The directorate did not seek to impose guidelines. It took time to consult with, and listen to the opinions of, the various partners. However, this type of management by consensus was ill-suited to a crisis, when it became critical to be able to make decisions quickly.

Second, information systems were unfit for logistics operations. These systems were originally developed with a view to financial reporting. For instance, the products coming out of the regional warehouse of a healthcare institution is considered an expense, although the product may be physically stored in a care unit. This shortcoming makes it more difficult to know the actual stock conditions in a given hospital. Also, these information systems are not able to produce consumption forecasts. And finally, each healthcare institution has its own product nomenclature (eg, product numbers), making it complex to get an exact snapshot of stocks in healthcare institutions.

Third, relations with suppliers were mostly limited to transactions. Quebec’s regulatory framework in this area may be the most complex among Canadian provinces. In 2008, Quebec adopted a law and regulations on contracts for public organizations, which included some 250 articles. That number has now more than doubled. This framework sets parameters for the contractual process and the nature of relationships with suppliers. Given this context, relationships between healthcare institutions and suppliers tend to be highly transactional in nature.

Lastly, embryonic performance management systems were in place. Research by Beaulieu and Roy showed that there was no real performance management system for logistics activities in healthcare institutions prior to the system’s reform.^13^ Generally, very few performance indicators are used in healthcare institutions’ logistics departments, and when they are, they are used at a very low frequency rate. The logistics departments of healthcare institutions are often reactive to any disruptions that may arise in the course of day-to-day operations.

## Recommendations

In the light of these observations, we offer some recommendations based on best practices, in order to be better prepared for future crises.

1. Identify predictors to anticipate the emergence of future pandemics: One Quebec hospital made a massive PPE purchase in mid-January, anticipating the effects of the pandemic (see Table 1). This institution took note of early warning signs, for example, the geographic spread of the virus combined with its mode of transmission, that were visible to all. In fact, pandemics can be anticipated, especially in North America, which tends to be the last place in which they gain a foothold.^14^ However, the monitoring of early pandemic signs should be done at the provincial level. There should be a provincial authority in charge of this. In the specific context of health, two teams could possibly take on this responsibility: public health decision makers or logistics decision makers. In theory, public health decision makers should be the ones monitoring for the potential emergence of a pandemic. However, it is difficult for public health departments to predict how a pandemic might impact on the supply of products and supplies, such as PPE. The Quebec experience shows that these decision makers primarily base their decisions on scientific evidence from multiple validated sources. Quebec’s public health department issued an initial advisory on COVID-19 just as the window of opportunity for implementing an effective logistics contingency plan was closing. However, supply chain decision makers are accustomed to making predictions and weighting risks. These approaches would make it possible to propose preventive measures such as stocking up on products and launching an operational pandemic management team.
2. Develop procurement expertise: This is required to make purchases that consider the particular characteristics and challenges of international markets and to analyze the risks associated with supply sources. During the pandemic, it was necessary to negotiate directly with foreign manufacturers, and very few buyers from the public sector were familiar with the specific regulations involved in international procurement.
3. Define a centralized logistics-management structure and allocation policies for a pandemic or other crisis: A centralized structure would simplify communications between suppliers and healthcare institutions, thereby reducing competition within the system. Personal protective equipment allocation policies must be regularly updated to reflect the current situation. We are not calling for a permanent centralized structure; however, the experience in Quebec and elsewhere confirms that such a structure is necessary in times of crisis.^15^
4. Ensure that key information for a demand-management system that’s indispensable during a pandemic is kept up to date: It is simpler to maintain this basic information from day to day than to try to update it in the midst of a crisis. Until this crisis, logistical information management was not a priority in Quebec’s health sector, and a great deal of effort was required to overcome this shortcoming during the pandemic.
5. Maintain close collaboration between logistics decision makers and the infection prevention and control departments in healthcare institutions: This kind of collaboration would give logistics managers more legitimacy when it comes to ensuring respect for PPE user protocols, and expertise for quality control in purchasing. The institutions that were able to quickly implement this type of collaboration avoided pointless purchases of the wrong products and were able to control consumption within their institution.
6. Improve collaboration between healthcare institutions and suppliers: Such collaboration can give suppliers more insight into demand, so that they can suggest solutions in the event of shortages. In return, suppliers could share strategic information on markets and their constraints with healthcare institutions. For Quebec’s healthcare sector, such a collaborative approach is a radical change of philosophy in terms of supplier management.

**Figure 2.**
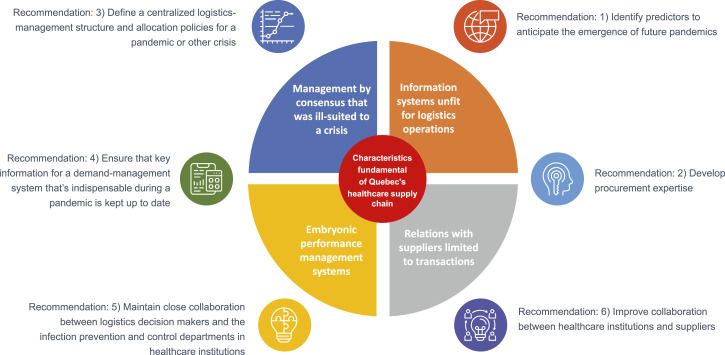
Summary of recommendations.

Figure 2 makes a link between the recommendations formulated and the basic features of Quebec’s health sector, which were presented in the previous section. As stated earlier, some of these features are not shortcomings as such but do hinder effective crisis management.

One fundamental challenge intersects with several of the formulated recommendations: breaking down organizational silos to foster communication between logistics decision makers, clinical decision makers, public health decision makers, and suppliers. This communication must not be thought of exclusively in terms of information technology but should encompass other integrative mechanisms (multidisciplinary teams, diverse professional profiles, and more).

Last, these recommendations fall mostly under the heading of preparedness measures, which do not prevent crises but rather minimize their management challenges.^16^ Preventive measures constitute a first-level response to help managers concentrate their efforts into finding solutions to unexpected problems once a crisis occurs. Furthermore, public decision makers should not limit their focus to only preparedness measures. Even though PPE management in Quebec was the starting point for these recommendations, many of them also address long-standing shortcomings in the logistics chain that are also present in many other jurisdictions in Canada and in other industrialized countries.^17^ Public deciders should support investments into new information technology and into skills training and development for the logistics staff of health establishments, and they should implement new practices with suppliers. The idea here is not only to prevent a new procurement crisis but also to enhance the day-to-day performance of the logistics chain. This is a real challenge that will require a great deal of perseverance, since the logistics chain for healthcare is possibly the most complex of all.

## References

[1] Beaulieu M Bentahar O Benzidia S . The evolution of healthcare logistics: the Canadian experience. J Appl Bus Econ. 2020;22(14):196-202.

[2] Cuellar AE Gertler PJ . Trends in hospital consolidation: the formation of local systems. Health Aff. 2003;22(6):77-87. 10.1377/hlthaff.22.6.77 14649434

[3] Boyer T . Le modèle Kaiser Permanente comme fondement de la réforme du système québécois de la santé et des services sociaux : que faut-il en retenir? Revue CNRIS. 2015;6(3). https://oraprdnt.uqtr.uquebec.ca/pls/public/gscw031?owa_no_site=357&owa_no_fiche=233.

[4] Landry S Beaulieu M Roy J . Strategy deployment in healthcare services: a case study approach. Technol Forecast Soc Change. 2016;113(B):429-437.

[5] Beaulieu M . Logistique hospitalière : performance des activités de gestion de l’approvisionnement des établissements québécois. Montréal: Centre sur la productivité et de la prospérité; 2019.

[6] Beaulieu M Rebolledo C . Repenser la performance de son groupe d’achats. In: Bentahar O Benzidia S , eds. Supply Chain Management de la santé. Caen, FR: Éditions Management & Société; 2019:359.

[7] Arney L Yadav P Miller R Wilkerson T . Strategic contracting practices to improve procurement of health commodities. Glob Health Sci Pract. 2014;2(3):295-306. 25276589 10.9745/GHSP-D-14-00068 PMC4168627

[8] Nollet J Beaulieu M Fabbe‐Costes N . Measuring purchasing groups performance in the health care sector. Can J Adm Sci. 2019;36(4):514-526.

[9] Bown CP . COVID-19: China’s exports of medical supplies provide a ray of hope. Available at: https://www.piie.com/blogs/trade-and-investment-policy-watch/covid-19-chinas-exports-medical-supplies-provide-ray-hope. Published March 26, 2020.

[10] De Montigny P . Le Canada est-il autosuffisant en équipement de protection individuelle? Available at: https://ici.radio-canada.ca/nouvelle/1731924/autosuffisance-canada-masques-equipement-protection-individuelle. Published October 7, 2020

[11] Torsekar M . Medtech’s supply chain: a decade in review. Available at: https://www.mpo-mag.com/issues/2020-01-29/view_columns/medtechs-supply-chain-a-decade-in-review/. Published January 29, 2020.

[12] Wadhwa P . Global annual spend on PPE can hit $50-80bn; China key player: Jefferies. Business Standard. Available at: https://www.business-standard.com/article/current-affairs/global-annual-spend-on-ppe-can-hit-50-80bn-china-key-player-jefferies-120063000576_1.html. June 3, 2020.

[13] Beaulieu M Roy J . Benchmarking de la gestion des achats et des stocks en milieu hospitalier : une démarche canadienne. Logistique Manag. 2015;23(3):17-27.

[14] Saunders-Hastings PR Krewski D . Reviewing the history of pandemic Influenza: understanding patterns of emergence and transmission. Pathogens. 2016;5(4):66. 27929449 10.3390/pathogens5040066 PMC5198166

[15] Bohmer RMJ Pisano GP Sadun R Tsai TC . How hospitals can manage supply shortages as demand surges. Harvard Business Review. Available at: https://hbr.org/2020/04/how-hospitals-can-manage-supply-shortages-as-demand-surges. April 3, 2020.

[16] Natarajarathinam M Capar I Narayanan A . Managing supply chains in times of crisis: a review of literature and insights. Int Jnl Phys Dist Log Manage. 2009;39(7):535-573.

[17] Srivastava S Garg D Agarwal A . A step towards responsive healthcare supply chain management: an overview. In: Singari RM Mathiyazhagan K Kumar H , eds. Advances in Manufacturing and Industrial Engineering. Lecture Notes in Mechanical Engineering. Singapore: Springer; 2021.

